# Reduced plasma levels of RGM-A predict stroke-associated pneumonia in patients with acute ischemic stroke: A prospective clinical study

**DOI:** 10.3389/fneur.2022.949515

**Published:** 2022-09-16

**Authors:** Jiaju Zhong, Juan Liao, Rongrong Zhang, Chanjuan Zhou, Zhenyu Wang, Siyuan Huang, Dan Huang, Mengliu Yang, Lei Zhang, Yue Ma, Xinyue Qin

**Affiliations:** ^1^Department of Rehabilitation Medicine, Yongchuan Hospital of Chongqing Medical University, Chongqing, China; ^2^Department of Neurology, The First Affiliated Hospital of Chongqing Medical University, Chongqing, China; ^3^Department of Central Laboratory, Yongchuan Hospital of Chongqing Medical University, Chongqing, China; ^4^Department of Endocrinology, The Second Affiliated Hospital, Chongqing Medical University, Chongqing, China

**Keywords:** repulsive guidance molecule A, ischemic stroke, pneumonia, inflammation, immunomodulation, prediction

## Abstract

**Background:**

Stroke-induced immunodepression syndrome is considered the major etiology of stroke-associated pneumonia (SAP). Repulsive guidance molecule A (RGM-A) is an immunomodulatory protein that is closely related to inflammation and immune responses. To explore the relationship between RGM-A and SAP and facilitate the early identification of patients at high risk of developing SAP, we investigated the predictive value of RGM-A in SAP.

**Methods:**

We enrolled 178 patients with acute ischemic stroke (AIS) and finally analyzed 150 patients, among whom 69 had SAP and 81 had non-SAP. During the same period, 40 patients with community-acquired pneumonia and 40 healthy participants were included as controls. SAP was defined according to the modified US Centers for Disease Control and Prevention criteria. Blood samples were collected at 24 h, 48 h, 3 days, 4 to 7 days, and 8 to 14 days after stroke onset. An enzyme-linked immunosorbent assay was used to detect the plasma levels of RGM-A and interleukin-6.

**Results:**

The plasma RGM-A levels were significantly decreased in both patients with community-acquired pneumonia and those with AIS, and the decline was most pronounced in patients with SAP (*P* < 0.001). RGM-A started to decline within 24 h after stroke in the SAP group, and the lowest levels were detected on day 3 and days 4 to 7 (*P* < 0.001). The RGM-A levels in the SAP group were lower than those in the non-SAP group at all blood collection time points (*P* < 0.05). In the logistic regression analyses, RGM-A was a protective factor for SAP after adjusting for confounders (adjusted odds ratio = 0.22, 95% confidence interval = 0.091–0.538, *P* = 0.001). Receiver operating characteristic curve analysis showed that the area under the curve for RGM-A was 0.766 (0.091–0.538; *P* = 0.001), the cutoff value was 4.881 ng/mL, and the sensitivity and specificity were 80.00 and 76.36%, respectively.

**Conclusions:**

We demonstrated that reduced plasma levels of RGM-A might help in the early identification of high-risk patients with SAP and predict the occurrence of SAP in patients with AIS. RGM-A might provide new clues to a potential alternative therapy for SAP.

## Introduction

Stroke is the second leading cause of death globally (1, 2). Stroke-associated pneumonia (SAP) is the most common complication after stroke and is significantly associated with death (3, 4), and with the incidence varying from 6.7 to 47% (5–9). SAP not only worsens stroke outcomes but also prolongs hospitalization and increases the economic burden on families and society (2, 5). Initial concepts on the etiology of SAP were mainly focused on aspiration, and a variety of studies have focused on age, stroke severity, stroke volume, and dysphagia in patients with SAP (4, 10). However, both nasogastric tubes and prophylactic antibiotic use have failed to reduce the incidence of SAP and the stroke mortality rate (10–12). The failure of these clinical trials has caused researchers to reconsider the etiology and therapeutic strategies of SAP. In recent years, accumulating experimental and clinical studies have confirmed that stroke-induced immunodepression syndrome (SIDS) is the main cause of SAP. Acute stroke causes a rapid and persistent deterioration in cellular immune function, inducing decreases in lymphocytes and natural killer cells and deactivation of monocytes, Th1 cells, and Th-mediated lymphocytes (3, 13, 14); this weakens the resistance of the human body against pathogens and leads to an increased susceptibility to SAP. Along these lines, immunomodulation has been explored as an alternative therapy for the prevention of SAP. However, highly sensitive and specific immunodepression biomarkers that can predict the occurrence of SAP are still lacking.

Repulsive guidance molecule A (RGM-A) is a 33-kDa glycosylphosphatidylinositol-linked membrane glycoprotein. It is the first molecule found to guide axons to their final location through a balance of chemoattractive or chemorepulsive signals in the developing nervous system (15–17). Recent evidence has identified RGM-A as a versatile immunoregulatory protein that is involved in a variety of inflammatory and immune diseases including autoimmune encephalomyelitis (18), multiple sclerosis (19), cerebrovascular atherosclerosis (20), peritonitis (21, 22), and acute lung injury (23, 24). RGM-A is strongly expressed in peripheral tissues, especially the surface of immune cells and tissues sensitive to infection, and it plays crucial roles in the regression of acute inflammation and tissue regeneration *via* its receptor neogenin (21, 22). *In vitro*, RGM-A restores MΦ chemotaxis and enhances macrophage phagocytosis, inhibiting polymorphonuclear leukocyte (PMN) migration (21, 22). *In vivo*, systemic application of RGM-A peptides was shown to attenuate the inflammatory response and infiltration of inflammatory cell traffic in a mouse model of zymosan-A-induced peritonitis (21, 22). In other studies involving an acute lung injury model, the RGM-A receptor neogenin was strongly induced within injured pulmonary tissue, and the binding of RGM-A to its receptor inhibited leukocyte migration, decreasing the production of proinflammatory cytokines and promoting inflammation resolution in acute lung injury (23, 24). Moreover, in our previous study, we found that RGM-A was involved in vascular inflammation and cerebrovascular atherosclerosis (20, 25). RGM-A levels were reduced in atherosclerotic aortas and in macrophages isolated from plaques (20).

Therefore, we hypothesized that RGM-A is associated with the development of SAP. To test this hypothesis, we designed the current study to investigate whether RGM-A is associated with SAP in patients with acute ischemic stroke (AIS). We found that reduced RGM-A levels might facilitate early prediction of the occurrence of SAP.

## Methods

### Patient selection

In this prospective clinical study, we recruited all patients with AIS from the Department of Neurology at Yongchuan Hospital of Chongqing Medical University from July 2020 to February 2022. During the same registration period, 40 healthy participants and 40 patients with community-acquired pneumonia (CAP) were recruited from the Physical Examination Center and Respiratory Department, respectively. This research was approved by the Ethics Committee of Yongchuan Hospital of Chongqing Medical University. All participants involved in this study provided written informed consent.

The inclusion criteria for patients with AIS were an age of ≥18 years, acute stroke onset within 72 h, confirmation of AIS by cranial computed tomography and magnetic resonance imaging at admission, and provision of informed consent. The exclusion criteria were a National Institutes of Health Stroke Scale (NIHSS) score of <1; active infection or pyrexia within 2 weeks before admission; cancer or autoimmune disease; unstable conditions such as renal failure, hepatic failure, heart failure, or immunosuppressant treatment; and loss to follow-up within 3 months. SAP was defined according to the modified United States Centers for Disease Control and Prevention criteria (26). SAP was diagnosed by two experienced neurologists during the first 7 days after the onset of stroke. CAP was defined as an acute lower respiratory infection associated with clinical signs and symptoms according to the British Thoracic Society (27), and the diagnosis of CAP was confirmed by pulmonary infiltrates on a chest computed tomography scan and laboratory indicators of acute lower respiratory infection at the time of hospitalization (28, 29).

**Figure 1:**
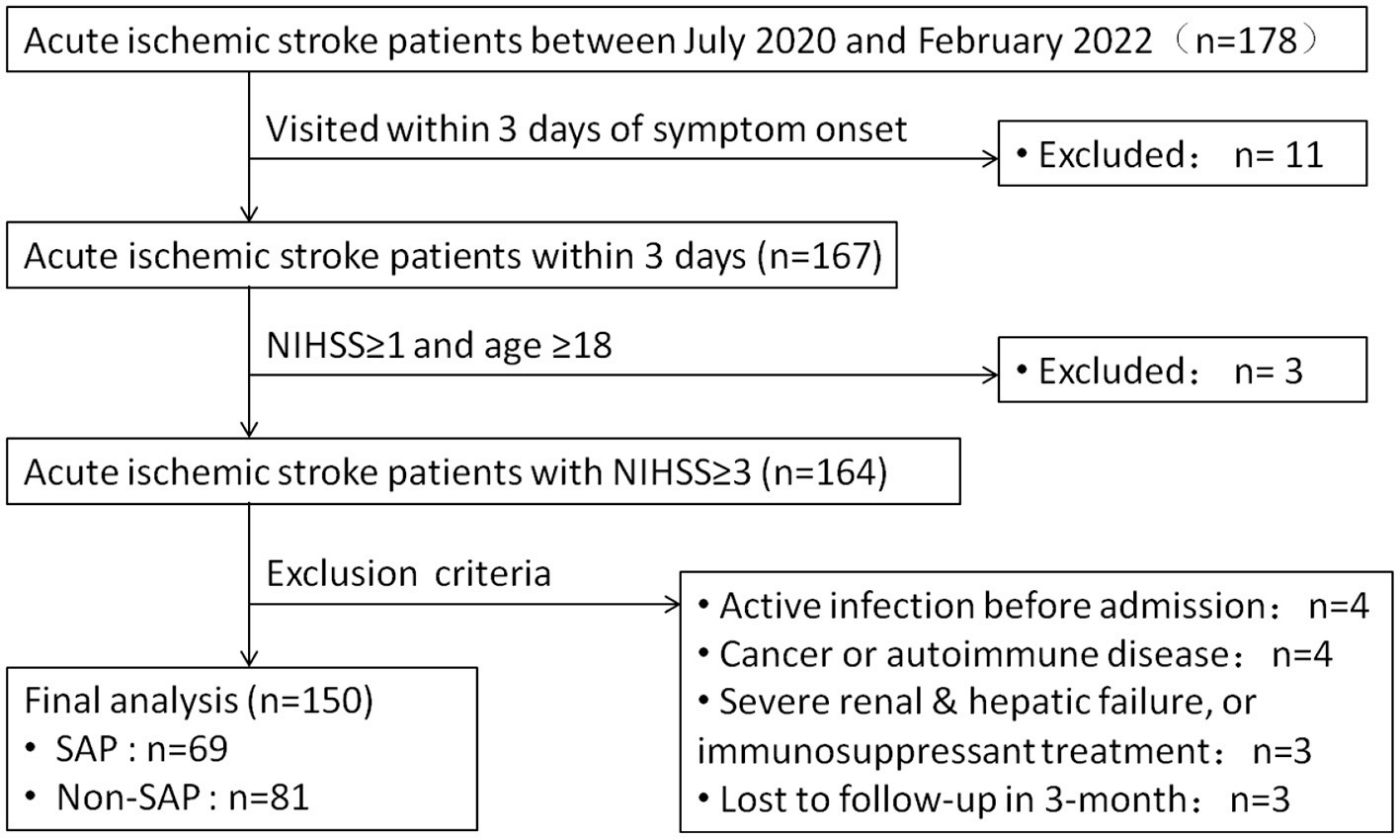
Flow-chart of inclusion and exclusion criteria. SAP, stroke-associated pneumonia; NIHSS, National Institutes of Health Stroke Scale.

In total, 178 patients with AIS were enrolled, and 28 were excluded. The remaining 150 patients with AIS were analyzed, among whom 69 had SAP and 81 had non-SAP (Figure 1).

### Clinical data

The baseline demographic and clinical data analyzed in this study were age, sex, and preexisting comorbidities including a history of smoking and drinking, hypertension, diabetes mellitus, hyperlipidemia, coronary heart disease, atrial fibrillation, previous stroke, and chronic obstructive pulmonary disease. Clinical parameters included the admission NIHSS score, the admission Glasgow Coma Scale score, dysphagia, infarct volume, and Trial of Org 10172 in Acute Stroke Treatment (TOAST) criteria. The NIHSS scores were divided into three categories: mild impairment (NIHSS score of 1–4), moderate impairment (NIHSS score of 5–15), and severe impairment (NIHSS score of ≥16) (13). Dysphagia was identified using bedside non-instrumented swallowing tests at admission. Glasgow Coma Scale assessment involves evaluation of eye-opening, motor, and verbal responses to speech. The etiological subtype of ischemic stroke was described according to the TOAST criteria. Large stroke volume was defined as an infarction involving two-thirds or more of the middle cerebral artery territory or internal carotid artery regions (30). All patients were followed up within 3 months after stroke onset. The follow-up was conducted by two experienced neurologists *via* a structured telephone interview to assess the modified Rankin Scale (mRS) score. A good outcome was defined as an mRS score of <3, and a poor outcome was defined as an mRS score of ≥3 (3–6), including death (31). The 30-day mortality rate and hospitalization duration were also included in the outcome assessment.

### Laboratory data

Fasting blood samples were collected at 24 h, 48 h, 3 days, 4 to 7 days, and 8 to 14 days after stroke onset. Because some patients were discharged or died within 14 days, blood samples were not collected at a fixed time point for every patient. Blood samples were collected in a calcium ethylenediaminetetraacetic acid tube (5 mL), and then centrifuged at low temperature and high speed (450×*g*, 4°C, 10 min). The plasma was collected and stored at −80°C for future use (31, 32). The plasma RGM-A and interleukin (IL)-6 concentrations were detected in accordance with the instructions for the enzyme-linked immunosorbent assay kit (R&D Systems, Minneapolis, MN, USA; 4 A BIOTECH, Beijing, China), and the absorbance value was detected with a multifunctional fluorescent luminescence analyzer (Varioskan Flash; Thermo Fisher Scientific, Waltham, MA, USA).

### Statistical analysis

Categorical variables were expressed as a number (%), and continuous variables with a normal distribution were expressed as the mean ± standard deviation. Data that did not fit a normal distribution were expressed as the median and interquartile range. Multiple group comparisons among the four different groups were made using the Kruskal-Wallis test. Differences in continuous variables between patients with and without SAP were analyzed using the chi-square test, Mann-Whitney U-test, or Kruskal-Wallis test, as appropriate. To investigate the association of RGM-A with SAP, adjusted odds ratios (aOR) and 95% confidence intervals (CIs) were calculated. Logistics regression analysis was used to analyze the predictive value of different variables for SAP. The diagnostic value of RGM-A was evaluated using the receiver operating characteristic (ROC) curve and the area under the ROC curve (AUC). Sensitivity and specificity were given under the maximal Youden's Index (sensitivity+specificity−1). Statistical analyses were performed using SPSS version 26.0 (SPSS Inc., Chicago, IL, USA). *P* < 0.05 was considered statistically significant, and *P* < 0.01 was considered highly statistically significant. Graphs were drawn using Graph Pad Prism 8 (Graph Pad Software, San Diego, CA, USA).

## Results

### Participant characteristics and clinical data

After recruitment, we preliminarily enrolled 178 patients with AIS in this study. Of these 178 patients, 28 were excluded (symptom onset >3 days previously, *n* = 11; NIHSS score of <1, *n* = 3; active infection within 2 weeks before admission, *n* = 4; cancer and autoimmune disease, *n* = 4; renal failure and hepatic failure, *n* = 3; and loss to follow-up within 3 months, *n* = 3) (Figure 1). The remaining 150 patients with AIS were analyzed, among whom 69 (46%) patients were assigned to the SAP group and 81 (54%) to the Non-SAP group. We also included 40 patients with CAP and 40 healthy subjects as controls.

As shown in Table 1, there were no differences in sex (*P* = 0.316), hypertension (*P* = 0.642), diabetes mellitus (*P* = 0.915), hyperlipidemia (*P* = 0.545), and chronic obstructive pulmonary disease (*P* = 0.177) between the SAP group and Non-SAP group. However, the patients' age and incidence of atrial fibrillation, coronary heart disease, and previous stroke were higher in the SAP group than in the Non-SAP group (*P* < 0.05). The incidence of severe neurological deficits (NIHSS score of ≥16), Glasgow Coma Scale score, dysphagia, infarct volume, and TOAST criteria were significantly different between the two groups (*P* < 0.01). Moreover, we evaluated the clinical outcomes of patients in the SAP and Non-SAP groups according to the 3-month mRS score, 30-day mortality, and hospitalization duration (Table 1). These results showed that the incidence of poor outcomes (3-month mRS score of 3–6) was significantly higher in the SAP group than in the Non-SAP group (75.4 vs. 32.1%, respectively; *P* < 0.001); similar results were observed for the 30-day mortality rate (29.0 vs. 3.7%, respectively; *P* < 0.001) and hospitalization duration (13.67 vs. 11.56 days, respectively; *P* < 0.05).

**Table 1:** Characteristics and clinical data of patients with AIS with and without SAP.

	**Non–SAP**	**SAP**	**χ^2^/t/z**	***P*–value**
*n* (%)	81 (54)	69 (46)		
Demographic parameters				
Age (SD), years	68.74 ± 11.60	75.04 ± 10.27	3.495	<0.001
Male, (*n* %)	50 (61.7)	37 (53.6)	1.005	0.316
Comorbidities				
Hypertension, (*n* %)	58 (71.6)	47 (68.1)	0.216	0.642
Diabetes mellitus, (*n* %)	24 (29.6)	21 (30.4)	0.012	0.915
Hyperlipidemia, (*n* %)	65 (80.2)	58 (84.1)	0.367	0.545
Atrial fibrillation, (*n* %)	22 (27.2)	32 (46.4)	5.972	0.015
Coronary artery disease, (*n* %)	20 (24.7)	32 (46.4)	7.736	0.005
Previous stroke, (*n* %)	8 (9.9)	16 (23.2)	4.913	0.027
COPD, (*n* %)	8 (9.9)	12 (17.4)	1.821	0.177
Clinical parameters				
Admission NIHSS score			20.918	<0.001
1–4, (*n* %)	24 (29.6)	6 (8.7)		
5–15, (*n* %)	46 (56.8)	33 (47.8)		
≥16, (*n* %)	11 (13.6)	30 (43.5)		
GCS score(IQR)	15 (13.5–15)	13 (10–15)	3.73	<0.001
dysphagia, (*n* %)	25 (30.9)	40 (58.0)	11.149	0.001
Large stroke volume, (*n* %)	45 (55.6)	62 (89.9)	21.436	<0.001
TOAST criteria			15.107	0.004
Large–artery atherosclerosis, (*n* %)	28 (34.6)	31 (44.9)		
Cardioembolism, (*n* %)	23 (28.4)	31 (44.9)		
Small–vessel occlusion, (*n* %)	23 (28.4)	6 (8.7)		
Other cause, (*n* %)	2 (2.5)	0 (0)		
Undefined cause, (*n* %)	5 (6.2)	1 (1.4)		
Outcomes				
3–month mRS			27.941	<0.001
good outcome (0–2), (*n* %)	55 (67.9)	17 (24.6)		
poor outcome (3–6), (*n* %)	26 (32.1)	52 (75.4)		
30–day mortality, (*n* %)	3 (3.7)	20 (29.0)	18.345	<0.001
Hospitalization duration (SD), days	11.56 ± 13.67	13.67 ± 7.70	2.067	0.04
Inflammatory predictors				
CRP (IQR), mg/L	1.40 (0.90–5.70)	14.80 (6.45–38.30)	6.632	<0.001
IL−6 (IQR), ng/mL	28.76 (21.48–37.44)	41.20 (29.29–92.00)	3.026	0.002
WBC (SD),10^∧^9/L	7.96 ± 2.84	10.38 ± 4.31	4.043	<0.001
NLR, (*n* %)	3.65 (2.24–8.14)	7.16 (4.84–12.10)	3.965	<0.001
NEUT, (*n* %)	69.84 ± 16.57	78.51 ± 13.75	3.379	0.001
Immunodepression marker				
RGM–A (SD), ng/mL	6.33 ± 2.02	4.94 ± 1.40	4.813	<0.001

Data are presented as mean ± standard deviation (SD), median (interquartile range, IQR), or *n* (%). AIS, acute ischemic stroke; SAP, stroke–associated pneumonia; COPD, chronic obstructive pulmonary disease; NIHSS, National Institutes of Health Stroke Scale; GCS, Glasgow Coma Scale; TOAST, Trial of Org 10172 in Acute Stroke Treatment; mRS, modified Rankin Scale; CRP, C–reactive protein; IL–6, interleukin–6; WBC, white blood cell; NLR, neutrophil–to–lymphocyte ratio; NEUT%, percentage of neutrophils; RGM–A, repulsive guidance molecule A.

We also analyzed laboratory data including the C-reactive protein (CRP) level, interleukin-6 (IL-6) level, neutrophil-to-lymphocyte ratio (NLR), white blood cell (WBC) count, and percentage of neutrophils (NEUT%) in patients with and without SAP (Table 1). We found that all included inflammatory predictors were substantially increased in patients with SAP and were significantly higher than those in patients with Non-SAP (*P* < 0.01): the CRP level was 14.80 (6.45–38.30) vs. 1.40 (0.90–5.70) mg/L, respectively (*P* < 0.001); the IL-6 level was 41.20 (29.29–92.00) vs. 28.76 (21.48–37.44) ng/mL, respectively (*P* = 0.002); the WBC count was 10.38 ± 4.31 vs. 7.96 ± 2.84 × 109/L, respectively (*P* < 0.001); the NLR was 7.16 (4.84–12.10) vs. 3.65 (2.24–8.14), respectively (*P* < 0.001); and the NEUT% was 78.51 ± 13.75% vs. 69.84 ± 16.57%, respectively (*P* = 0.001). These results suggest that elevated inflammatory factors were associated with the occurrence of SAP and that the diagnosis of SAP in this study was credible. Notably, the RGM-A level was significantly lower in the SAP group than in the Non-SAP group (4.94 ± 1.40 vs. 6.33 ± 2.02, respectively; *P* < 0.001), indicating that a relationship might exist between RGM-A and SAP. Therefore, we next investigated the possibility of this relationship.

### Plasma RGM-A levels were significantly decreased in patients with CAP and AIS

**Figure 2:**
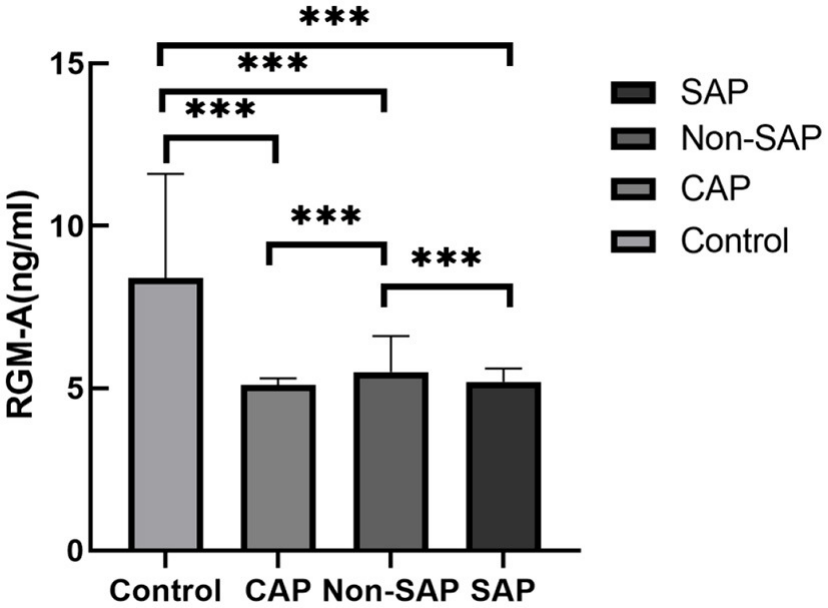
Comparison of plasma RGM-A levels among the Control, CAP, Non-SAP, and SAP groups.RGM-A, repulsive guidance molecule A; CAP, community-acquired pneumonia; SAP, stroke-associated pneumonia. ***P < 0.001.

To further investigate the relationship between RGM-A and SAP, we included 40 patients with CAP and 40 healthy subjects as controls (Table 2). First, we found that the RGM-A levels were significantly lower in patients with CAP than in healthy controls [5.1 (5.1–5.3)c vs. 8.4 (6.5–11.6) ng/mL, respectively; *P* < 0.001] (Table 2, Figure 2). These results suggest that RGM-A is involved in the acute inflammatory reaction in the lung. In addition, the RGM-A levels were significantly lower in patients who had AIS without SAP than in healthy controls [5.5 (5.2–6.6) vs. 8.4 (6.5–11.6) ng/mL, respectively; *P* < 0.001]. Furthermore, among patients with AIS, these decreases were more pronounced in patients with than without SAP (5.2 [3.7–5.6] vs. 5.5 [5.2–6.6] ng/mL, respectively; *P* < 0.001). These results indicate that RGM-A is not only associated with acute lung infection but is also related to AIS, especially in patients with SAP.

**Table 2:** Baseline characteristics and RGM–A levels among the four groups.

	**Control (*n* = 40)**	**CAP (*n* = 40)**	**Non–SAP (*n* = 81)**	**SAP (*n* = 69)**	***P*-value**
Age (SD), years	64.0 ± 11.2	69.6 ± 13.5 ^a^	68.7 ± 11.6 ^a^	75.0 ± 10.3^a, b, c^	0.001
Male, (*n* %)	22(55.0)	28 (70.0)	50 (61.7)	37 (53.6)	0.344
Current smoking, (*n* %)	11 (27.5)	12 (30.0)	34 (42.0)	25 (36.2)	0.371
Current drinking, (*n* %)	10 (25.0)	14 (35.0)	21 (25.9)	19 (27.5)	0.721
Hypertension, (*n* %)	0 (0)	17 (42.5)	58 (71.6)	47 (68.1)	<0.001
Diabetes mellitus, (*n* %)	0 (0)	4 (10.0)	24 (29.6)	21 (30.4)	<0.001
Hyperlipidemia, (*n* %)	0 (0)	3 (7.5)	65 (80.2)	58 (84.1)	0.013
Systolic pressure (SD), mmHg	124.9 ± 18.6	130.2 ± 23.1	157.3 ± 27.3^a, b^	153.7 ± 32.1^a, b^	<0.001
Diastolic pressure (IQR), mmHg	72.0 (66.3–80.0)	72.0 (65.0–84.0)	90.0(77.0–98.5)^a, b^	87.0 (75.0–97.0)^a, b^	<0.001
RGM–A(IQR), ng/mL	8.4 (6.5–11.6)	5.1 (5.1–5.3)^a^	5.5 (5.2–6.6)^a, b^	5.2 (3.7–5.6)^a, c^	<0.001

Data are presented as mean ± standard deviation (SD), median (interquartile range, IQR), or *n* (%). RGM–A, repulsive guidance molecule A; CAP, community–acquired pneumonia; SAP, stroke–associated pneumonia. ^a^*P* < 0.05 for CAP, Non–SAP, SAP vs. Control; ^b^*P* < 0.05 for Non–SAP, SAP vs. CAP; ^c^*P* < 0.05 for SAP vs. Non–SAP.

### Time course of RGM-A and inflammatory predictors in SAP and Non-SAP groups

**Figure 3:**
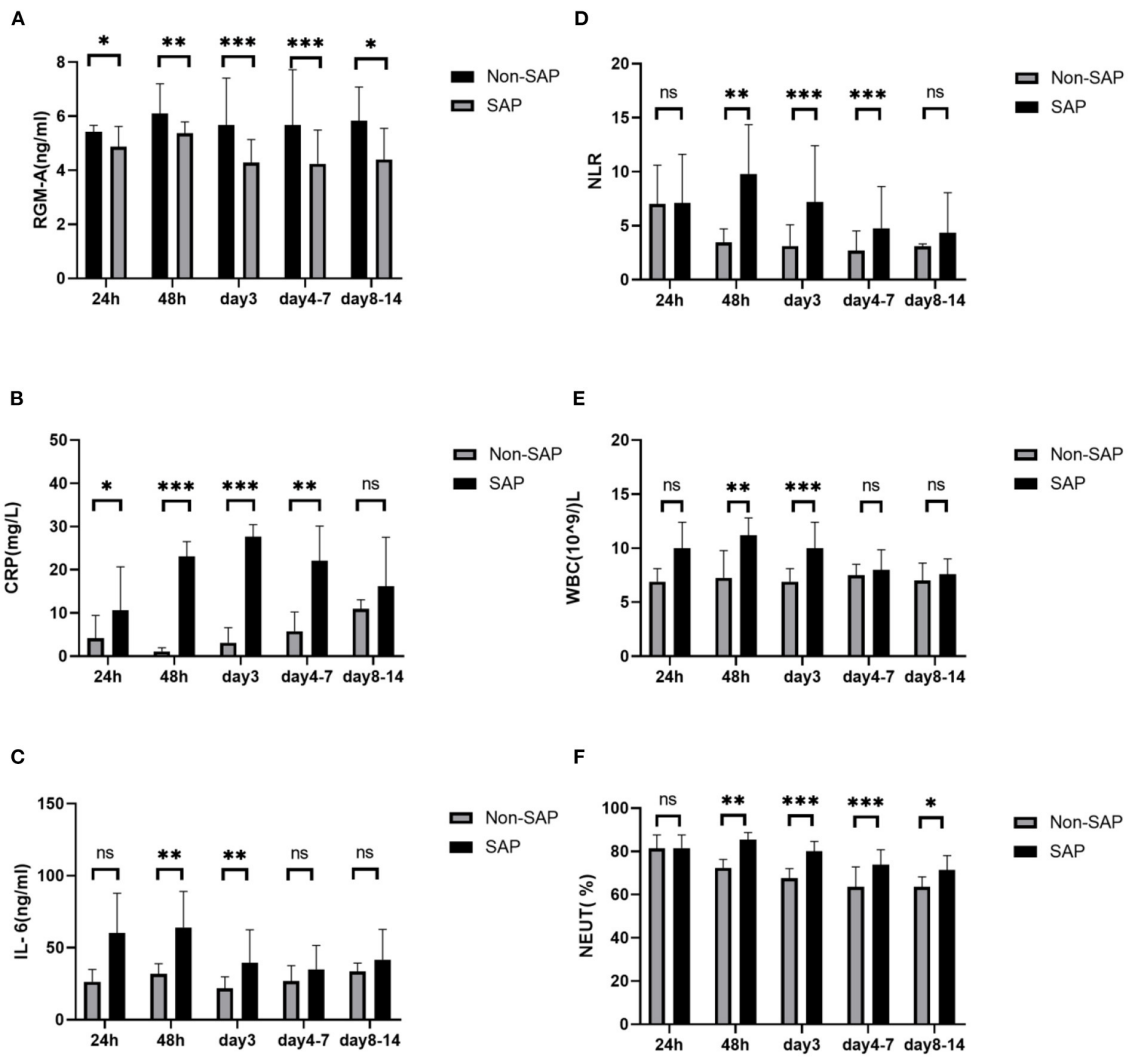
Levels of RGM-A and inflammatory predictors at 24 h, 48 h, 3 days, 4 to 7 days, and 8 to 14 days after stroke onset in patients with and without SAP. **(A)** RGM-A level, **(B)** CRP level, and **(C)** IL-6 level in patients with SAP and Non-SAP, **(D)** NLR, **(E)** WBC count, and **(F)** NEUT% in patients with SAP and Non-SAP. SAP: stroke-associated pneumonia; RGM-A: repulsive guidance molecule A; CRP: C-reactive protein; IL-6: interleukin-6; WBC: white blood cell; NLR: neutrophil-to-lymphocyte ratio; NEUT%: percentage of neutrophils. *P < 0.05, **P < 0.01, and ***P < 0.001; ns, non-significant (*P* > 0.05).

To investigate the changes of RGM-A over time, we collected fasting blood samples at 24 h, 48 h, 3 days, 4 to 7 days, and 8 to 14 days after stroke onset. Figure 3A shows that the RGM-A expression started to decline during the initial 24 h after stroke in the SAP group (*P* < 0.05), and the lowest levels were reached on day 3 and days 4 to 7 (*P* < 0.001). These differences between the SAP and Non-SAP groups tended to shrink on days 8 to 14 (*P* < 0.05). Moreover, the RGM-A levels in the SAP group were lower than those in the Non-SAP group at all time points (*P* < 0.05) (Figure 3A). These results might mean that reduced RGM-A levels are associated with the development of SAP.

Furthermore, with the exception of the CRP level (*P* < 0.05) (Figure 3B), the other inflammatory predictors (IL-6 level, NLR, WBC count, and NEUT%) did not change significantly within 24 h after stroke (*P* > 0.05) (Figures 3B–F). The NLR and NEUT% were significantly increased on day 2, day 3, and days 4 to 7 in the SAP group (*P* < 0.01) (Figures 3C,E). The IL-6 level and WBC count were increased only on day 2 and day 3 (*P* < 0.01) (Figures 3D,F) and began to decrease on days 4 to 7 (*P* > 0.05) (Figures 3D,F). Finally, all the inflammatory predictors (CRP level, IL-6 level, NLR, WBC count, and NEUT%) were decreased on days 8 to 14 (Figures 3C–F). Based on these findings, we speculate that the variations of inflammatory predictors might be related to the disease process and antibiotic use in patients with SAP.

### Logistic regression analysis

To further understand the risk factors for SAP, we used logistic regression to analyze the predictive value of different confounding variables for SAP (Table 3). The results showed that RGM-A was a protective factor for SAP after adjusting for confounders (adjusted odds ratio: aOR = 0.221, 95% confidence interval: CI = 0.091–0.538, *P* = 0.001), whereas the CRP level (aOR = 1.157, 95% CI = 1.06–1.264, *P* = 0.001) and WBC count (aOR = 1.236, 95% CI=1.051–1.45, *P* = 0.009) were risk factors. Consistent with our hypothesis, RGM-A might be a protective factor for SAP, and patients with reduced plasma RGM-A levels may be more likely to develop SAP.

**Table 3:** Multivariable analysis of possible predictors of SAP.

**Variable**	**β**	**S.E**	**aOR (95% CI)**	***P*-value**
RGM–A	−1.51	0.454	0.221 (0.091–0.538)	0.001
CRP	0.146	0.045	1.157 (1.06–1.264)	0.001
WBC	0.212	0.081	1.236 (1.051–1.45)	0.009
GCS	−0.246	0.105	0.782 (0.636–0.961)	0.019

Logistic regression analysis was performed to analyze the predictive value of different confounding variables for SAP. β, β-regression coefficient; S.E, standard error; aOR, adjusted odds ratio; CI, confidence interval; RGM–A, repulsive guidance molecule A; CRP, C–reactive protein; WBC, white blood cell; GCS, Glasgow Coma Scale.

### Receiver operating characteristic (ROC) analysis

ROC analysis was performed to evaluate the diagnostic value of RGM-A and other conventional inflammatory indicators for SAP (Table 4). The results showed that the AUC for RGM-A was 0.766 (95% CI: 0.686–0.847; *P* < 0.001), which was higher than that for IL-6 [0.758 (0.640–0.876); *P* = 0.0002], NLR [0.714 (0.629–0.799); *P* < 0.001], WBC [0.709 (0.622–0.796); *P* < 0.001], and NEUT% [0.696 (0.61–0.783); *P* < 0.001] but slightly lower than the AUC for CRP [0.839 [0.761–0.916]; *P* < 0.001). The cutoff value for RGM-A was 4.881 ng/mL, and the sensitivity and specificity were 80.00 and 76.36%, respectively. These results indicated that RGM-A had a good diagnostic value for SAP.

**Table 4:** Comparison of predictive power of RGM–A vs. conventional inflammatory indicators in the prediction of SAP.

**Variable**	**AUC**	***P*-value**	**95% confidence interval**		**Cutoff value**	**Sensibility, %**	**Specificity, %**
RGM–A	0.766	*P* <0.001	0.686	0.847	4.881, ng/mL	80.00	76.36
CRP	0.839	*P* <0.001	0.761	0.916	9.050, mg/L	79.16	82.19
IL–6	0.758	0.0002	0.64	0.876	32.557, ng/mL	72.97	74.19
NLR	0.714	*P* <0.001	0.629	0.799	4.225	55.42	84.21
WBC	0.709	*P* <0.001	0.622	0.796	7.90, 10^∧^9/L	54.67	78.26
NEUT	0.696	*P* <0.001	0.61	0.783	77.10,	57.74	79.45

Receiver operating characteristic analysis was performed to evaluate the diagnostic value of RGM–A and other conventional inflammatory indicators for SAP. AUC, area under the curve; RGM–A, repulsive guidance molecule A; CRP, C–reactive protein; IL–6, interleukin–6; WBC, white blood cell; NLR, neutrophil–to–lymphocyte ratio; NEUT%, percentage of neutrophils.

## Discussion

In this study, we focused on identifying potential associations between the plasma RGM-A level and the development of SAP, while also investigating the changes of RGM-A over time. We found that a reduced plasma RGM-A level increased the risk of SAP in patients with AIS, and the earliest decline started as early as 24 h after stroke onset. We believe that a reduced plasma RGM-A level might help in the early identification of high-risk patients with SAP.

Stroke is an acute neurovascular disease with high morbidity, mortality, and disability rates (2). SAP is one of the most serious complications after stroke and is significantly associated with poor outcomes (3). Early identification of patients at high risk for SAP might help prevent the onset of SAP and ameliorate its consequences. Several studies have focused on age, stroke severity, stroke volume, and dysphagia in patients with SAP, and several clinical predictive models have been established to select patients at high risk for SAP (3, 4, 10, 33, 34). However, highly sensitive and specific immunodepression biomarkers that can predict the occurrence of SAP are still lacking.

To our knowledge, this study is the first to show that the plasma RGM-A level is decreased in patients with SAP. RGM-A is a promising target for the diagnosis and treatment of numerous diseases based on its versatility; it is involved in ischemic stroke (35), Parkinson's disease (36), autoimmune encephalomyelitis (18), multiple sclerosis (19), and cerebrovascular atherosclerosis (20). The evaluation of RGM-A as an immunoregulatory protein has recently increased in translational medical research, especially with regard to peritonitis (21, 22) and acute lung injury (23, 24). In our previous study, we found that the RGM-A level was reduced in atherosclerotic aortas and in macrophages isolated from plaques, suggesting that RGM-A has a potential protective effect in cerebrovascular atherosclerosis (20). Previous researchers have demonstrated that RGM-A is expressed in pathogenic Th17 cells in experimental autoimmune encephalomyelitis (18), and the binding of RGM-A to its receptor neogenin inhibits PMN migration and increases the attachment of CD4+ cells to intercellular adhesion molecule-1 to attenuate the inflammatory response (18, 21, 22). RGM-A restores MΦ chemotaxis and enhances macrophage phagocytosis, inducing human MΦ toward alternatively activated (M2) MΦs. One study showed that administering RGM-A peptides attenuated the inflammatory response and infiltration of inflammatory cell traffic in a mouse model of zymosan-A-induced peritonitis (21, 22). In recent years, researchers have found that RGM-A and its receptor neogenin are involved in the acute inflammatory response in pulmonary tissue (23). The binding of RGM-A to its receptor neogenin was shown to inhibit leukocyte migration, decrease the production of proinflammatory cytokines, and promote inflammation resolution in acute lung injury (23, 24). In the absence of RGM-A, neogenin has a damaging effect on lung tissue, resulting in PMN entry into the inflammatory site of lung tissue and exacerbation of the inflammatory response (37). However, when RGM-A binds to its receptor neogenin, it exerts anti-inflammatory activity to block the migration of PMNs to the inflammatory site (21, 22). Therefore, we are reasonably confident that the plasma RGM-A level is associated with the development of SAP.

Moreover, we found that the expression of RGM-A was decreased in patients with AIS (patients in the Non-SAP group). One explanation for the close relationship between the RGM-A and AIS may be the immunologic changes after stroke events. It is well known that stroke induces rapid and temporary immunodepression, inducing SIDS. SIDS leads to a decrease in the number of lymphocytes and natural killer cells as well as deactivation of monocytes, Th1 cells, and Th-mediated lymphocytes (14). RGM-A is a membrane-binding immunomodulatory protein that is closely related to inflammation and immune responses. It is highly expressed in immune cells and is cleaved and presents in soluble form in serum under inflammatory conditions (21, 22).When SIDS occurs, immune cells decrease and RGM-A expression might be reduced, which would explain the reduction of RGM-A in patients with AIS.

Although biomarkers such as serum iron (38), intercellular adhesion molecule 1 (32), procalcitonin (39), and human leukocyte antigen–DR isotype (13) have been investigated to predict the development of SAP, highly sensitive and specific immunodepression biomarkers that might help in the early identification of high-risk patients with SAP are still lacking. Notably, our study showed that RGM-A started to decline within 24 h after stroke onset, and the lowest levels were observed on day 3 and days 4 to 7. This might have occurred because SAP is an early complication and most frequently occurs within the first 7 days after stroke, and SIDS usually begins within several hours after stroke and is even more pronounced during the following 3 days (13, 40). Moreover, the expression of RGM-A rather than other inflammatory indicators, such as CRP and IL-6, continued decreasing 8 to 14 days after stroke, indicating that SIDS might last longer than the inflammatory response. This may explain why antibiotic prophylaxis fails to reduce the frequency of SAP or improve 3-month outcomes in patients with stroke (11, 12). Thus, these results might provide clues for a more effective exploration of alternative therapy for the prevention of SAP.

Our research has several limitations. First, SAP was diagnosed according to clinical symptoms, chest computed tomography findings, and inflammatory biomarkers, but sputum cultures often remain negative without bacterial growth. Second, the incidence of SAP in our study was higher than that in other reports in the literature. This might be related to the fact that we performed a single-center study with a relatively small sample size, and our stroke center often receives patients in quite poor condition from local areas. Therefore, multicenter studies should be carried out to expand the sample size and further verify the experimental results. Third, because some patients were discharged or died within 14 days, blood samples were not collected at a fixed time point for every patient. Finally, to better understand the relationship between RGM-A and SAP, future studies should evaluate the very early changes in RGM-A that occur within the first hours after stroke onset.

In conclusion, this study is the first to identify a relationship between RGM-A and SAP, and our results suggest that a reduced plasma RGM-A level might help in the early identification of high-risk patients with SAP. RGM-A might provide new clues to a potential alternative therapy for SAP.

## Data availability statement

The original contributions presented in the study are included in the article/Supplementary material, further inquiries can be directed to the corresponding author/s.

## Ethics statement

The studies involving human participants were reviewed and approved by the Ethics Committee of Yongchuan Hospital of Chongqing Medical University. The patients/participants provided their written informed consent to participate in this study. Written informed consent was obtained from the individual(s) for the publication of any potentially identifiable images or data included in this article.

## Author contributions

XQ and JZ participated in study design and study conception. JZ and JL performed data analysis and wrote the manuscript. RZ, CZ, ZW, SH, DH, and MY recruited patients and performed the laboratory analyses. XQ, JZ, LZ, and MY revised the manuscript. All authors provided critical review of the manuscript and approved the final draft for publication.

## Funding

This work was supported by the National Natural Science Foundation of China (grant numbers 81771275, 82071338, and 82101375) and the National Natural Science Foundation of Yongchuan District (grant numbers 2020nb0227).

## Conflict of interest

The authors declare that the research was conducted in the absence of any commercial or financial relationships that could be construed as a potential conflict of interest.

## Publisher's note

All claims expressed in this article are solely those of the authors and do not necessarily represent those of their affiliated organizations, or those of the publisher, the editors and the reviewers. Any product that may be evaluated in this article, or claim that may be made by its manufacturer, is not guaranteed or endorsed by the publisher.
