# Supplementary material for: Reduced plasma levels of RGM-A predict stroke-associated pneumonia in patients with acute ischemic stroke: A prospective clinical study
Source: Front Neurol. 2022 Sep 16;13:949515. doi: 10.3389/fneur.2022.949515 (PMC9523133; doi:10.3389/fneur.2022.949515)
Supplement: Supplementary file 1 [file Table_1.DOCX]

**Supplementary Materials**

**TABLE 1: Laboratory indicators in AIS Patients with and Without SAP**

|  | Non-SAP(n=81) | SAP(n=69) | χ^2^/t/z | P Value |
| --- | --- | --- | --- | --- |
| BUN (IQR), mmol/L | 5.72(4.94-7.20) | 6.31(5.28-8.00) | 1.966 | 0.050 |
| Cr (SD), umol/L | 73.52±33.44 | 78.69±40.55 | 0.804 | 0.423 |
| UA (SD), umol/L | 324.11±97.33 | 316.05±98.35 | 0.468 | 0.640 |
| ALT (IQR), U/L | 22.00(16.00-29.00) | 24.00(15.75-34.00) | 0.829 | 0.407 |
| AST (SD, U/L | 25.92±10.66 | 28.19±9.59 | 1.305 | 0.194 |
| TC (SD, mmol/L | 4.67±1.09 | 4.50±1.14 | 0.895 | 0.372 |
| TG (SD, mmol/L | 1.57±1.35 | 1.35±0.95 | 1.242 | 0.216 |
| HDL (SD, mmol/L | 1.35±0.32 | 1.43±0.71 | 0.902 | 0.369 |
| LDL (SD, mmol/L | 2.57±0.93 | 2.47±0.94 | 0.642 | 0.522 |

Note: Laboratory indicators in AIS Patients with and Without SAP in supplementary. Data are presented as mean ± standard deviation (SD), median (interquartile range, IQR). SAP: stroke-associated pneumonia; BUN: blood urea nitrogen; Cr: creatinine; UA: uric acid; ALT: alanine aminotransferase; AST: aspartate aminotransferase; TC: total cholesterol; TG: triglycerides; HDL: high-density lipoprotein; LDL: low-density lipoprotein. P>0.05 indicates no significant difference.

**TABLE 2: The levels of RGM-A and inflammation predictors at different time points in patients with and without SAP**

|  |  | RGM-A | CRP | IL-6 | NLR | WBC | NEUT% |
| --- | --- | --- | --- | --- | --- | --- | --- |
| 24h | non-SAP(n=38) | 5.43(5.22-5.66) | 4.20(1.37-9.45) | 33.95±19.98 | 7.02(2.89-10.61) | 9.22±3.57 | 75.98±17.19 |
|  | SAP(n=15) | 4.88(3.68-5.62) | 10.60(4.13-79.70) | 64.92±53.82 | 7.10(4.80-11.60) | 11.16±5.69 | 80.85±10.11 |
|  | t/z | 2.034 | 2.042 | 1.660 | 0.848 | 1.473 | 1.024 |
|  | p | 0.042 | 0.041 | 0.130 | 0.397 | 0.147 | 0.311 |
| 48h | non-SAP(n=18) | 6.97±2.52 | 1.50±1.09 | 31.63±9.46 | 4.68±3.72 | 7.76±2.16 | 71.66±8.94 |
|  | SAP(n=21) | 4.94±1.26 | 34.27±33.39 | 81.34±56.20 | 11.79±9.05 | 10.86±3.44 | 82.18±9.24 |
|  | t/z | 3.100 | 4.275 | 3.001 | 3.123 | 3.126 | 3.404 |
|  | p | 0.005 | ＜0.001 | 0.011 | 0.005 | 0.004 | 0.002 |
| Day 3 | non-SAP(n=36) | 6.51±1.83 | 3.10(1.10-6.60) | 21.82(15.11-29.80) | 3.13(2.11-5.09) | 7.27±1.91 | 66.21±11.05 |
|  | SAP(n=22) | 4.21±1.07 | 27.70(11.15-37.43) | 39.57(29.29-92.38) | 7.19(5.37-12.40) | 10.55±3.57 | 78.87±9.18 |
|  | t/z | 5.356 | 4.973 | 2.772 | 3.753 | 3.894 | 4.411 |
|  | p | ＜0.001 | ＜0.001 | 0.006 | ＜0.001 | ＜0.001 | ＜0.001 |
| Days 4-7 | non-SAP(n=45) | 5.67(5.16-7.72) | 5.75(3.10-16.23) | 26.79(23.74-37.52) | 2.70(2.05-4.52) | 7.87±1.68 | 64.96±10.54 |
|  | SAP(n=52) | 4.24(3.31-5.49) | 22.10(9.45-49.10) | 35.00(24.19-51.56) | 4.78(3.53-8.62) | 8.58±2.66 | 73.54±8.95 |
|  | t/z | 4.225 | 2.850 | 1.457 | 3.570 | 1.372 | 3.628 |
|  | p | ＜0.001 | 0.004 | 0.145 | ＜0.001 | 0.175 | 0.001 |
| Days 8-14 | non-SAP(n=17) | 6.30±2.79 | 10.95(1.18-13.05) | 33.46(22.58-39.28) | 3.11(2.28-3.32) | 7.00(4.30-8.63) | 62.18±6.74 |
|  | SAP(n=38) | 4.60±1.91 | 16.20(6.10-47.50) | 42.68(18.96-62.65) | 4.35(3.05-8.05) | 7.60(6.13-9.00) | 70.79±10.16 |
|  | t/z | 2.624 | 1.690 | 0.883 | 2.042 | 0.862 | 1.983 |
|  | p | 0.011 | 0.091 | 0.377 | 0.041 | 0.389 | 0.055 |

Note: Levels of RGM-A and inflammatory predictors at 24 hours, 48 hours, 3 days, 4 to 7 days, and 8 to 14 days after stroke onset in patients with and without SAP. Data are presented as mean ± standard deviation (SD), median (interquartile range, IQR). SAP: stroke-associated pneumonia; RGM-A: repulsive guidance molecule A; CRP: C-reactive protein; IL-6: Interleukin-6; WBC: white blood cell count; NLR: neutrophil-to-lymphocyte ratio; NEUT%: percentage of neutrophils. P>0.05 indicates no significant difference; P<0.05 indicates a statistically significant difference; P<0.01 indicates an extremely statistically significant difference.
